# Spontaneous second-trimester ruptured pregnancy of rudimentary horn: a case report in Yaounde, Cameroon

**DOI:** 10.11604/pamj.2014.18.86.4579

**Published:** 2014-05-26

**Authors:** Florent Ymele Fouelifack, Jovanny Tsuala Fouogue, John Owoudou Messi, Danielle Tiako Kamga, Jeanne Hortence Fouedjio, Zacharie Sando

**Affiliations:** 1 Obstetrics and Gynecology Unit of the Yaounde Central Hospital, Yaounde, Cameroon; 2 Department of Obstetrics and Gynecology of the Faculty of Medicine and Biomedical Sciences of the University of Yaounde 1, Yaounde, Cameroon; 3 Department of Anatomy and Morphological Sciences of Faculty of Medicine and Biomedical Sciences of the University of Yaounde 1, Yaounde, Cameroon

**Keywords:** Ectopic pregnancy, rudimentary uterine horn, Cameroon, rupture, emergency

## Abstract

Rudimentary uterine horn pregnancy is rare and, to our knowledge, has not been previously reported in Cameroon. We herein report the case of a 22 year old second gravida referred for acute abdominal pain at 17 weeks of gestation. Physical examination revealed hemoperitoneum with hypovolemic shock. After resuscitation, an emergency exploratory laparotomy was done and we found hemoperitoneum of 3,500 milliliters, a bicornuate uterus with a ruptured right rudimentary communicating horn containing a non viable foetus. There were no other abnormalities. We performed an excision of the rudimentary horn with ipsilateral salpingectomy. Post-operative course was uneventful and the woman was discharged seven days later. This case emphasizes the importance of good antenatal care to avoid complications.

## Introduction

Bicornuate uterus is a condition resulting from faulty unification of paramesonephric (Müllerian) ducts during intra-uterine life. It's encountered in 0.1-3% of females [1]. There is no causal relationship between Müllerian anomalies and infertility, but they are correlated with adverse pregnancy outcomes [2, 3]. Several cases of ruptured rudimentary uterine horn pregnancies have been reported, but the prevalence is not known [4–9]. We present a case managed in the Gyneco-Obstetric Unit of Yaounde Central Hospital.

## Patient and observation

**Figure 1:**
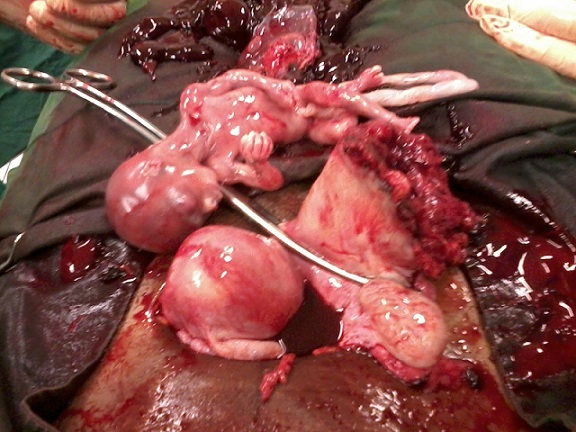
View of the operative field showing the non viable foetus and the ruptured rudimentary uterine horn

Miss EL is a 22 year old, G2P1001 student, referred to our emergency setting for an abdominal pain evolving since 12 hours, in a pregnancy of 17 weeks. That abdominal pain started abruptly 12 hours prior to consultation. It was diffuse, of moderate intensity and without irradiation. This motivated an unfruitful automedication with mebeverine. A sudden and sharp raise of intensity motivated a consultation in a health center from where she was immediately referred to out setting. The patient had her first menses at 14 years. She bleeds for 5 days every 30 days. She has never had neither dysmenorrhoea nor dyspareunia. She had no method of contraception. Her first pregnancy resulted in a normal delivery of a live baby boy weighing 3,450 grammes two years earlier. The current pregnancy was 17 weeks and was poorly followed up in a health center. Her medico - surgical and social past history was unremarkable. Clinical assessment on admission revealed a hemorrhagic shock characterized by: asthenia, vomiting, vertigo, restlessness, anxiety, a blood pressure of 80/55 millimeters of mercury, a pulse rate of 123 pulsations /minute, a respiratory rate of 28 cycles/minutes. The temperature was 37.3 degree Celsius. Conjunctivae were pale. Chest examination revealed tachycardia and polypnea. The abdomen was distended, symmetrical, tender and dull at the percussion. Bowel sounds were absent. Vaginal speculum revealed no abnormality. On digital vaginal exploration, the cervix was posterior, long and closed. Posterior cervical fornix was full and painful. Cervical motion tenderness was noted but bimanual exam was limited by pain. A paracentesis brought back 8 millimeters of non clotting blood. The working diagnosis was a ruptured ectopic pregnancy and the differential diagnosis was ruptured ovarian cyst in pregnancy. An emergency laparotomy was indicated and a preoperative workup done, showing a hemoglobin level of 6.4 grammes per deciliter. Resuscitation and monitoring were immediately started with mask oxygen, intravenous fluids, blood transfusion, in-dwelling Foley's catheter and painkillers. After informed consent, surgery was started three hours after admission. This delay was due technical (availability of blood and emergency medicines), financial (lack of money to buy emergency drugs) and human resource (small number of anesthetists on call duty at night) constraints. Operative findings were: a hemoperitoneum of 3,500 milliliters, a bicornuate uterus with a ruptured right rudimentary non-communicating horn containing a non viable foetus weighing 280 grammes and macroscopically normal fallopian tubes and ovaries (Figure 1). Excision of the rudimentary horn and ipsilateral salpingectomy were done and the patient received four pints of packed red blood cells. Uneventful post-operative course followed and the woman was discharged seven days later.

## Discussion

Rudimentary horn pregnancy is rare because of the rarity of the underlying mullerian malformation. It is associated with adverse pregnancy outcomes [1–3]. The prevalence of unicornuate uterus is estimated at 1/4,020 women in the general population [3]. To our knowledge only one case has been reported so far in Cameroon [10] Patients with müllerian anomalies usually present with pelvic pain, endometriosis, hematometria, hematocolpos, recurrent pregnancy loss, and preterm delivery [2]. They can also remain asymptomatic, till fortuitous discovery during imaging procedures for other purposes or during surgery as it was the case in our patient [2, 10].

Since ultrasonography highly depends on operator's skills, many cases of rudimentary uterine horn pregnancies are missed like in our case [4]. A skillful ultrasonography can detect some müllerian anomalies but confirmation requires magnetic resonance imaging [7, 8]. Though neonatal survival rate in rudimentary uterine horn is only 11%, a correct diagnosis during the first trimester ultrasound would have at least prevented maternal morbidity in our case [9]. Neonatal survival occurs in the following circumstances reported in literature: premature delivery and term rudimentary horn pregnancy [4, 9]. In our case, laparotomy started three hours after it was indicated. This tremendous delay is of course unacceptable but it is commonly noticed in our emergency unit and is attributable to institutional weaknesses.

Surgical management of ruptured rudimentary horn pregnancy involves excision of the uterine horn hosting the pregnancy and homolateral salpingectomy; this can be done via laparotomy of laparoscopy [5–7]. Before rupture, if the foetus is viable cesarean section followed by ablation of the rudimentary horn may be indicated because induction with misoprostol carries a high risk of rupture [4, 7]. Before foetal viability, non ruptured rudimentary horn pregnancy is best managed by surgery [8].

## Conclusion

Though rare, rudimentary horn pregnancy should be present in the mind of every practitioner facing an acute surgical abdomen in pregnant woman. Good quality antenatal care in first trimester can avoid complications like rupture.
